# PD-L1 expression and survival in p16-negative and -positive squamous cell carcinomas of the vulva

**DOI:** 10.1007/s00432-020-03126-9

**Published:** 2020-02-05

**Authors:** Bastian Czogalla, Deborah Pham, Fabian Trillsch, Miriam Rottmann, Julia Gallwas, Alexander Burges, Sven Mahner, Thomas Kirchner, Udo Jeschke, Doris Mayr, Elisa Schmoeckel

**Affiliations:** 1 Department of Obstetrics and Gynecology, University Hospital, Ludwig Maximilians University (LMU), Munich, Germany; 2 Institute of Pathology, Ludwig Maximilians University (LMU), Munich, Germany; 3 Munich Cancer Registry, Bavarian Cancer Registry-Regional Centre Munich (LGL) at the University Hospital of Munich, Institute for Medical Information Processing, Biometry and Epidemiology, Ludwig Maximilians University (LMU), Munich, Germany

**Keywords:** Squamous cell carcinoma of the vulva, PD-L1, p16, TILS

## Abstract

**Aim:**

Programmed death-ligand 1 (PD-L1) has become a widely used predictive biomarker for therapy with checkpoint inhibitors in a variety of cancers. Here, we studied the expression of PD-L1 in squamous cell carcinomas of the vulva (SCCV) with regard to HPV status via its surrogate marker p16. Additionally, the status of PD-L1 and p16 were analyzed for prognostic information and potential correlation to tumor-infiltrating lymphocytes (TILs).

**Methods:**

PD-L1 was analyzed in 128 cases of SCCV using the tumor proportion score (TPS), the immune cell score (ICS) and the combined positive score (CPS). Cases were immunostained for p16 and analyzed for stromal TILs. PD-L1, p16, and TILs were compared to clinico-pathological parameters and patient’s survival.

**Results:**

TPS ≥ 50% and CPS ≥ 50 were correlated to a worse grading (*p* = 0.028 and *p* = 0.031), but not to FIGO-stage. CPS ≥ 50 was associated to a worse prognosis with overall survival (*p* = 0.021) but was not correlated to the progression-free survival. P16-positivity was correlated to a longer progression-free survival (*p* = 0.006) and overall survival (*p* = 0.023). PD-L1 expression was independent from p16 status. TILs ≥ 50% were present in 24% of the cases and were strongly correlated to PD-L1 (TPS *p* = 0.02, ICS *p* < 0.001, CPS *p* = 0.001).

**Conclusion:**

Our data demonstrate that PD-L1 expression is frequent in SCCV and independent from p16 status. High PD-L1 expression was associated with an unfavorable outcome whereas p16-positivity turned out to be an independent positive prognostic factor.

**Electronic supplementary material:**

The online version of this article (10.1007/s00432-020-03126-9) contains supplementary material, which is available to authorized users.

## Introduction

Squamous cell carcinoma of the vulva (SCCV) is a relatively rare disease, accounting for 5% of all gynecologic malignancies (Siegel et al. 2016). However, incidence rates are increasing, particularly due to increase in younger women (Lai et al. 2014; Schuurman et al. 2013; Hampl et al. 2008). SCCV can be either human papillomavirus (HPV)-associated or -independent. Up to 25–40% are linked to HPV-infection and the other group to chronic inflammatory and degenerative skin diseases, particularly lichen sclerosus (Gargano et al. 2012; Del Pino et al. 2013). Compared to oropharyngeal squamous cell carcinoma, the prognostic impact of HPV is considerably less established in SCCV. However, indications increase that HPV-linked SCCV have a more favourable prognosis (Sand et al. 2019; Lee et al. 2016). Mainstay of the therapy is surgery, which can be accompanied by radiation and/or chemo-radiation. Furthermore, advanced-stages have limited treatment options and there are only a few clinical trials for vulvar cancer. Therefore, target-based therapies and predictive biomarkers are needed to improve the clinical outcome of recurrent or metastatic disease.

Checkpoint inhibitors are among the most promising therapeutic approaches, being effective in a variety of cancers by leading to a strong immune-response against tumor cells by blocking PD1 or PD-L1 (Lyford-Pike et al. 2013). Immunostaining for PD-L1 has become a valid predictive biomarker that is routinely analyzed in several types of cancer. So far single case reports could demonstrate that PD-L1 inhibitors might be useful in SCCV (Shields and Gordinier 2019; Ott et al. 2019). Regarding different tumor types studies are controversial, whether PD-L1 expression is a prognostic marker too (Troiano et al. 2019; Wang et al. 2017; Wang 2019).

Recent studies about oropharyngeal squamous cell carcinomas and cervical cancer of the uterus indicate that PD-L1 expression is related to HPV status, suggesting that PD-L1 expression is increased in HPV-associated carcinomas (Mezache et al. 2015, 2017; Badoual et al. 2013; Lyford-Pike et al. 2013). Here, we assessed the HPV status in SCCVs via its surrogate marker p16.

Tumor-infiltrating lymphocytes (TILs) are an indicator of the immunogenic surveillance of cancer. Several studies could demonstrate a correlation of increased PD-L1 expression and numbers of TILs suggesting that both factor are cooperative (Meng et al. 2018). Furthermore, high percentages of TILs are associated with a better prognosis in several types of cancer including gynecologic cancers (Ruffini et al. 2009; Xu et al. 2019; Shah et al. 2011; Meng et al. 2018). However they have been hardly studied in SCCV.

This study aimed to investigate the potential prognostic impact of PD-L1 in p16-negative and p16-positive SCCVs and putative associations with stromal TILs.

## Materials and methods

### Study group and clinical data

The study population was generated consecutively and included 128 cases of SCCV, treated between 1994 and 2008 at the Department of Obstetrics and Gynecology, Ludwig-Maximilians-University, Munich, Germany. All tissue samples were derived from surgical resections, biopsies were excluded from the study group. Patient’s age ranges from 20 to 96 (median age 71). Complete follow-up data were available for all cases with a median follow-up time of 66.7 months (standard deviation 58.9). 81 of the 128 patients (63%) died during the follow-up period.

### Immunohistochemistry

Immunohistochemical stains were performed using formalin-fixed paraffin-embedded (FFPE) tissues. To measure up to the heterogeneity of the PD-L1-staining, a whole tumor block was used for immunohistochemistry in each case. Sections were cut at 4 µm from each paraffin block and mounted on SuperFrost Plus microscope slides (Menzel Gläser, Braunschweig, Germany), deparaffinized and stained with hematoxylin and eosin (HE). Immunohistochemistry was then performed for PD-L1 (clone SP263, Ventana, ready-to-use) and p16 (clone E6H4E6H4/p16^Ink4a^, Ventana, ready-to-use). Immunohistochemistry was subjected to heat-induced epitope unmasking by heating with a pressure cooker and performed on a Ventana Benchmark XT autostainer (Ventana Medical Systems, Oro Valley, AZ) with the XT UltraView diaminobenzidine kit (Vector Laboratories, Burlingame, CA) and hematoxylin counterstaining (Vector Laboratories, Burlingame, CA). Positive controls were included.

### Evaluation of PD-L1, p16 and TILs

PD-L1 status was assessed using the Tumor proportion score (TPS) (Scheel et al. 2016), the Immune cell score (ICS) (Rosenberg et al. 2016) and the Combined positive score (CPS) (Agilent Dako 2018).

For the evaluation of PD-L1 expression in tumor cells, the TPS was used referring to 0 =  < 1%, 1 =  ≥ 1% and < 5%, 2 =  ≥ 5% and < 10%, 3 =  ≥ 10% and < 25%, 4 =  ≥ 25% and < 50% and 5 =  ≥ 50%. PD-L1 status in immune cells was evaluated by the ICS defined by ICS 0 < 1%; ICS 1 ≥ 1% and < 5%; ICS 2 ≥ 5% and < 10%; ICS 3 ≥ 10%. For evaluation of PD-L1 expression in both tumor cells and immune system, the CPS was used. The CPS is defined by the number of PD-L1 staining tumor cells and immune cells (lymphocytes, macrophages) divided by the total number of viable tumor cells, multiplied by 100. Although, the result of the calculation can exceed 100, the maximum score is defined as CPS 100. PD-L1 staining was considered positive if the cell membrane was partially or completely stained, irrespective of the staining intensity. Cytoplasmic PD-L1 staining was disregarded.

P16-positivity was defined by a strong cytoplasmic and nuclear staining throughout the whole tumor on slide (“block” staining). Cases showing a weak or patchy staining were considered p16-negative.

TILs were assessed by HE staining. In this study, we analyzed the “stromal” TILs, which are defined by immune cells within the stroma of the tumor but without direct contact to the tumor cells. According to guidelines of the “TILs working group”, the percentage of TILs covering the stroma of the tumor was estimated regarding the whole tumor-area on the slide (Salgado et al. 2015). The infiltration of TILs included lymphocytes, plasma cells and macrophages. Granulocyte-rich areas and necrosis were disregarded. First percentage of TILs were scored in 5%-steps and then dichotomized into TILs < 50% and ≥ 50%.

### Statistics

For statistical analysis, the SPSS Statistics version 23 (SPSS Inc., Chicago, IL, USA) and SAS 9.4 (SAS software, Cary, NC, USA) were used. For testing proportional differences in univariate analysis, the Pearson’s Chi-square test or the Fisher’s exact test for qualitative variables. The survival curves were generated using the Kaplan–Meier technique and differences between these curves were tested by the log-rank test. For multivariate analyses, the Cox regression model for progression-free and overall survival (PFS, OS) was used. All tests were two-sided and the level of statistical significance was accepted at *p ≤ *0.05.

### Ethics

All patients’ data were fully anonymized, and the study was performed, according to the standards set in the Declaration of Helsinki 1975. The tumor tissue used was leftover material that had initially been collected for histopathological diagnostics. All diagnostic procedures have already been fully completed when samples were retrieved for the study. The current study was approved in writing by the Ethics Committee of the Ludwig-Maximilians-University, Munich, Germany (approval number 19-261). Authors were blinded for clinical information during experimental analysis.

## Results

### Expression of PD-L1 correlates with clinico-pathological parameters

**Fig. 1:**
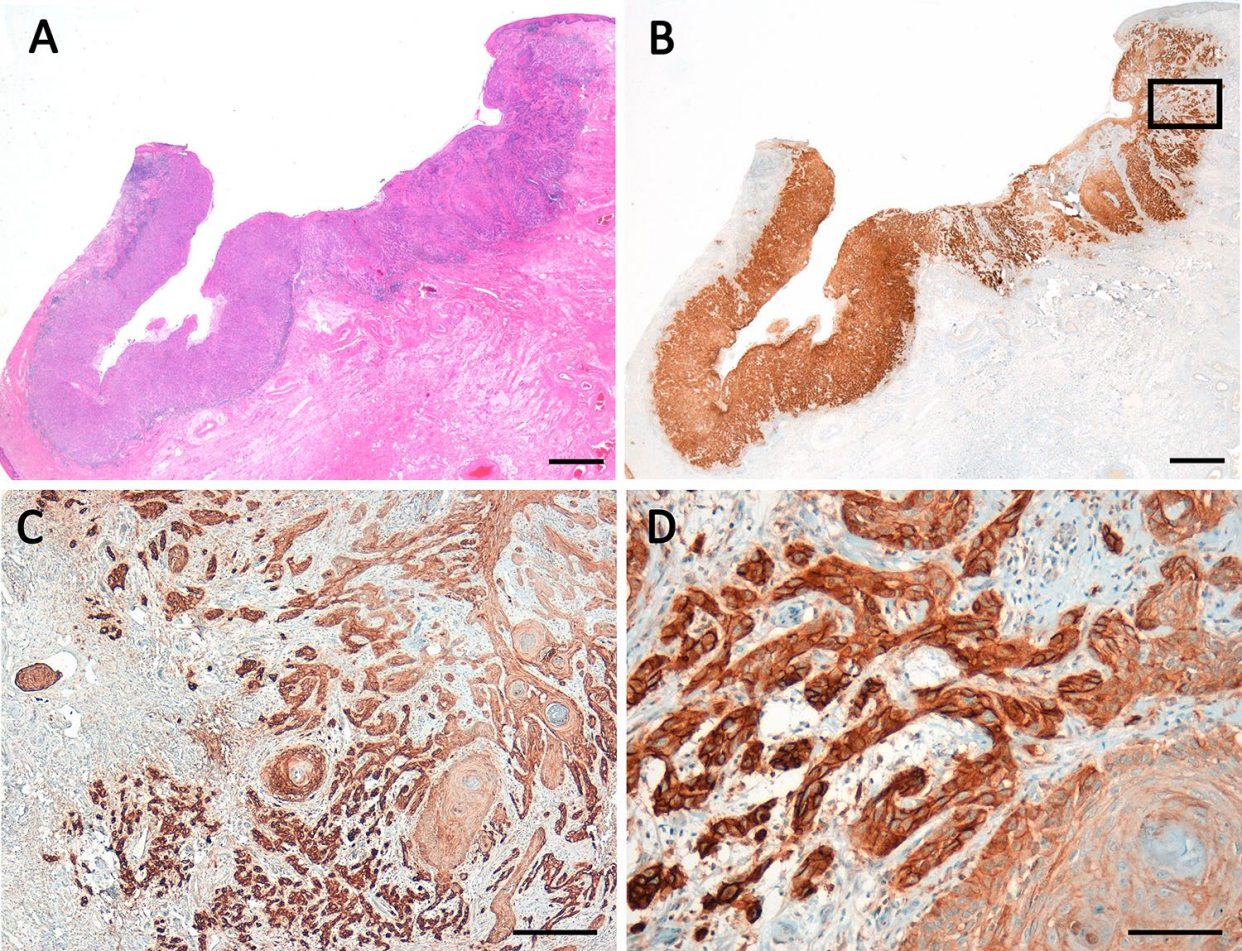
Squamous cell carcinoma of the vulva showing high expression of PD-L1 in the tumor cells (PD-L1-positivity in 95% of the tumor cells; TPS 5, CPS ≥ 100) and moderate expression of PD-L1 in stromal immune cells (ICS 2). **c** and **d** Refer to the inset in **b**. **a** HE, **b**–**d** PD-L1. Scale bars **a** and **b** 2.0 mm, **c** 200 µm, **d** 100 µm

**Fig. 2:**
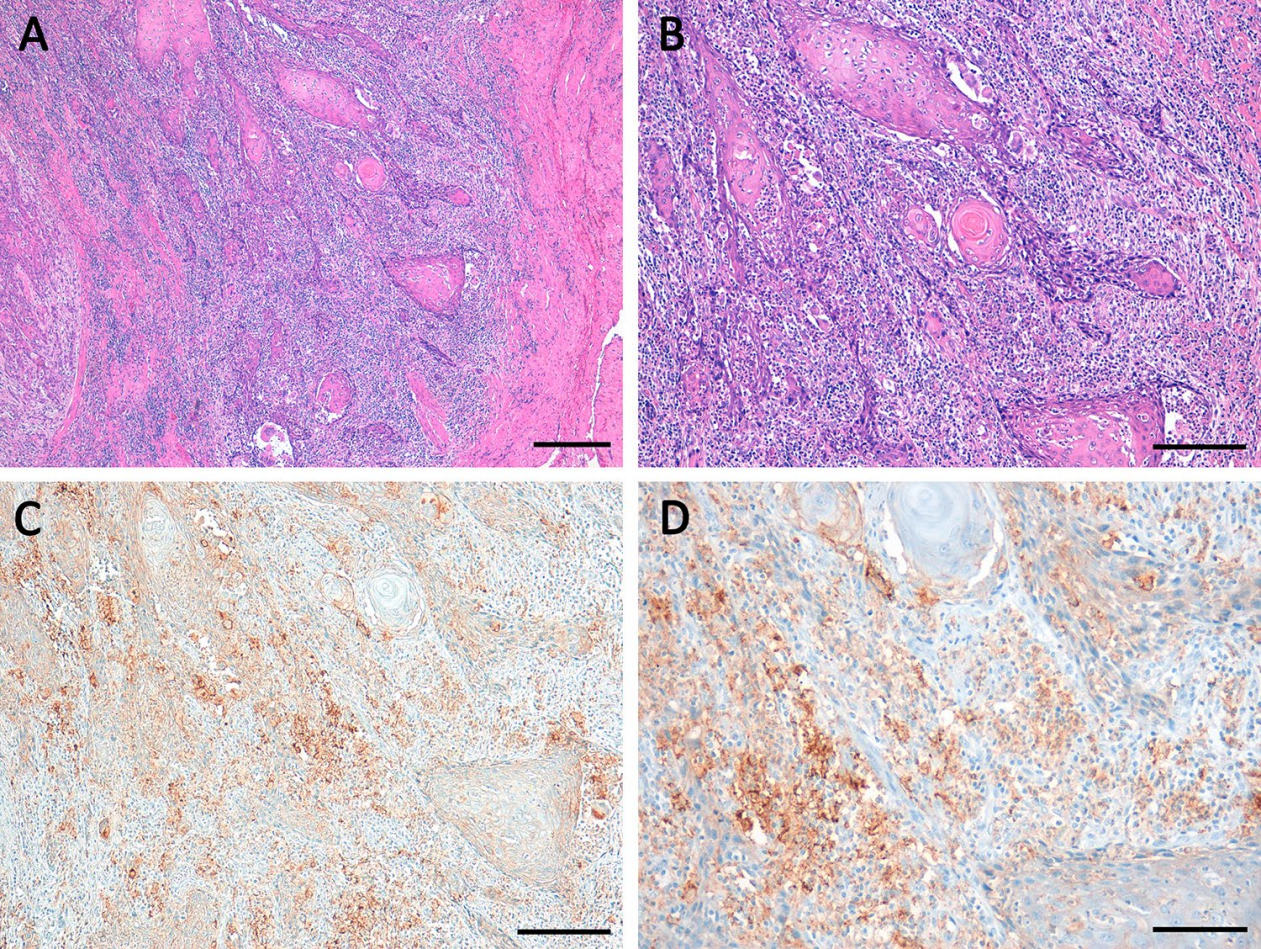
Squamous cell carcinoma of the vulva with high percentage of stromal TILs (TILs ≥ 50%) in **a** and **b** (HE) associated to high PD-L1 expression **c**, **d** in stromal immune cells (ICS 3) and moderate PD-L1 expression in tumor cells (TPS 4, CPS ≥ 50 < 80). Scale bars **a** 400 µm; **b** and **c** 200 µm; **c** 100 µm

PD-L1 positivity was observed in the majority of the cases: TPS ≥ 1% was found in 83%, ICS ≥ 1% in 93%, and CPS ≥ 10 in 66%. About 15% of the cases showed a high expression of PD-L1, defined by TPS ≥ 50% and CPS ≥ 50. TPS ≥ 50% was significantly correlated to ICS ≥ 10% (*p* = 0.026) and CPS ≥ 50 (*p* < 0.001). High PD-L1 expression (TPS ≥ 50% and CPS ≥ 50) was significantly correlated to a worse grading (*p* = 0.031 resp. *p* = 0.033). However, there was no correlation to the FIGO-stage. Results of the evaluation of TPS, ICS, and CPS are listened in Table 2. The PD-L1 profile in association to the clinico-pathological parameters are listed in Tables 1 and 2 and illustrated in Figs. 1 and 2.

**Table 1:** P16-positivity (p16 +), TILs ≥ 50% and high PD-L1 expression (TPS 5, ICS 3 and CPS ≥ 50) in correlation to patients characteristics and the overall survival (*n* = 128)

Variables	Total (%)	p16 +	TILs ≥ 50%	PD-L1 TPS 5 (≥ 50%)	PD-L1 ICS 3 (≥ 10%)	PD_L1 CPS ≥ 50
Total (%)		50 (39)	31 (24)	24 (19)	57 (45)	25 (20)
**Age**						
< 70 years	57 (45)	32 (64)	16 (52)	14 (58)	25 (44)	14 (56)
≥ 70 years	71 (55)	18 (36)	15 (48)	10 (42)	32 (56)	1 1 (44)
* p* value	–	**< 0.001**	0.362	0.131	0.891	0.198
**Grading**						
G1	17 (13)	3 (6)	4 (13)	0 (0)	9 (16)	0 (0)
G2	77 (60)	33 (66)	21 (68)	14 (58)	37 (65)	15 (60)
G3	34 (27)	14 (28)	6 (19)	10 (42)	11 (19)	10 (40)
* p* value	–	0.149	0.550	**0.028**	0.233	**0.031**
**FIGO**						
FIGO I	36 (28)	18 (36)	12 (39)	5 (21)	16 (28)	6 (24)
FIGO II	52 (41)	19 (38)	11 (36)	9 (38)	23 (40)	8 (32)
FIGO III	31 (24)	9 (18)	7 (23)	9 (38)	13 (23)	10 (40)
FIGO IV	9 (7)	4 (8)	1 (3)	1 (4)	5 (9)	1 (4)
* p* value	–	0.338	0.426	0.373	0.992	0.225
**Overall survival (univariate analysis)**						
* p* value		**< 0.001**	0.130	0.465	0.599	0.133

**Table 2:** PD-L1 status of the study group (TPS, ICS and CPS)

**TPS**	**0 (< 1%)**	**1 (≥ 1%)**	**2 (≥ 5%)**	**3 (≥ 10%)**	**4 (≥ 25%)**	**5 (≥ 50%)**
*N* (%)	26 (17.0)	17 (11.1)	21 (13.7)	19 (12.4)	21 (13.7)	24 (15.7)
**CPS**	**0 – < 10**	**10 – < 50**	**50 – < 80**	**80 – 100**		
*N* (%)	52 (34.0)	51 (33.3)	21 (13.7)	4 (2.6)		
**ICS**	**0 (< 1%)**	**1 (≥ 1%)**	**2 (≥ 5%)**	**3 (≥ 10%)**		
*N* (%)	11 (7.2)	29 (19)	31 (20.3)	57 (37.3)		

### Expression of PD-L1 is correlated with overall survival

Prognostic information was only evident in analysis with CPS while TPS and ICS did not provide significant results. High PD-L1 expression according to CPS ≥ 50 was significantly correlated to a worse prognosis in multivariate Cox regression analysis with OS (*p* = 0.021; Table 3). However, CPS ≥ 50 was not significant in univariate analysis (*p* = 0.133; Table 1). CPS ≥ 50 was not correlated to the PFS (*p* = 0.190 for univariate and *p* = 0.157 for multivariate analyses; Table 3).

Focusing on p16-negative cases only (*n* = 78) CPS ≥ 50 showed a trend to potentially shorter OS in multivariate Cox regression analysis (*p* = 0.071).

**Table 3:** Multivariate Cox regression analysis with (a) overall survival and (b) progression-free survival for CPS (*n* = 128)

Variables	*p* value	Hazard ratios	95% Confidence interval
**(a) Overall survival**			
Age (< 70 versus ≥ 70)	< 0.001	0.255	0.148–0.440
Grading (G1 versus G2/3)	0.413	0.744	0.366–1.510
FIGO (I versus II–IV)	0.005	0.415	0.225–0.766
CPS (< 50 versus ≥ 50)	0.021	0.535	0.314–0.910
**(b) Progression-free survival**			
Age (< 70 versus ≥ 70)	0.378	0.779	0.447–1.358
Grading (G1 versus G2/3)	0.704	1.161	0.537–2.511
FIGO (I versus II–IV)	0.417	0.777	0.422–1.431
CPS (< 50 versus ≥ 50)	0.157	0.626	0.327–1.197

### Status of p16 correlates with clinico-pathological parameters and affects the progression-free and overall survival

**Fig. 3:**
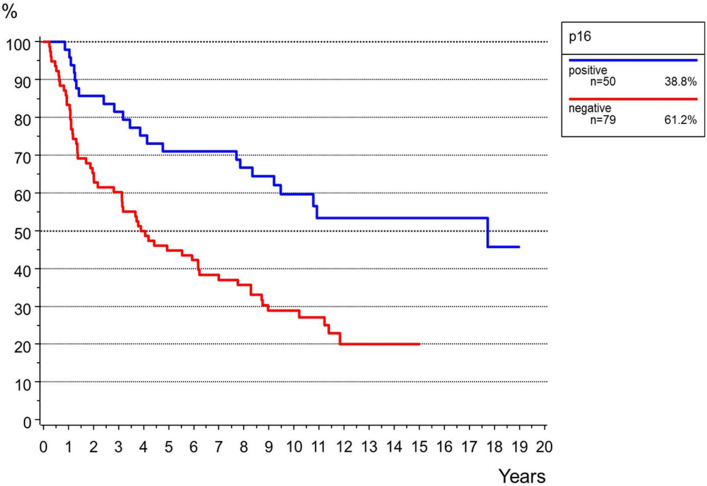
Overall survival for the status of p16 (*n *= 128, *p* < 0.001)

P16-positivity was found in 50 (39%) and p16-negativity in 78 cases (61%) (Supplementary Fig. 1). P16-positivity was significantly correlated to the patient’s age (*p* < 0.001) but not to FIGO-stage or grading (Table 1). P16-positivity was significantly correlated to a longer PFS and OS in both univariate (PFS *p* = 0.004, OS *p* < 0.001) and multivariate analyses (PFS *p* = 0.006, OS *p* = 0.023; Table 4, Fig. 3, Supplementary Fig. 2).

**Table 4:** Multivariate Cox regression analysis with (a) overall survival and (b) progression-free survival for p16 (*n* = 128)

Variables	*p* value	Hazard ratio	95% Confidence interval
**(a) Overall survival**			
Age (< 70 versus ≥ 70)	< 0.001	0.339	0.195–0.589
Grading (G1 versus G2/3)	0.139	0.590	0.293–1.186
FIGO (I versus II–IV)	0.006	0.420	0.227–0.776
p16 (positive versus negative)	0.023	0.550	0.329–0.920
**(b) Progression-free survival**			
Age (< 70 versus ≥ 70)	0.844	1.059	0.600–1.859
Grading (G1 versus G2/3)	0.728	0.874	0.409–1.868
FIGO (I versus II–IV)	0.486	0.805	0.437–1.482
p16 (positive versus negative)	0.006	0.408	0.215–0.771

Status of p16 was independent of PD-L1 expression regarding TPS 5 (*p* = 0.290), ICS 3 (*p* = 0.591) and CPS ≥ 50 (*p* = 0.176).

### Stromal TILs are associated with expression of PD-L1

Stromal TILs ≥ 50% were found in 31 cases (24%; Table 1, Fig. 2). TILs ≥ 50% were significantly associated to a high PD-L1 expression using all three scores: *p* = 0.02 for TPS, *p* < 0.001 for ICS 3, and *p* = 0.001 for CPS ≥ 50. Stromal TILs ≥ 50% were not correlated to FIGO-stage, grading; patients age or to patients’ overall survival (Table 1) nor to the status of p16 (*p* = 0.394).

## Discussion

We herein report that PD-L1 positivity is a frequent finding in SCCV. The majority of the study population showed a weak to moderate PD-L1 immunoreactivity in tumor cells and immune cells (TPS ≥ 1% in 83% and ICS ≥ 1% in 93% of the study group). PD-L1 expression in tumor cells was concordant to the expression in immune cells. TPS was significantly correlated to the ICS (*p* = 0.026) and strongly to the CPS (*p* < 0.001). High PD-L1 expression (TPS ≥ 50% and CPS ≥ 50) was observed in about 15% and was associated with a worse grading, but was independent from FIGO-stage and was also found in early cancer stages.

Little is known about the PD-L1 status in SCCV, but high frequency of PD-L1 expression was also reported by a few other studies (Choschzick et al. 2018; Hecking et al. 2017; Thangarajah et al. 2019). Currently clinical data about checkpoint-inhibitor therapy in SCCV are limited, although responsiveness was reported for single cases (Shields and Gordinier 2019; Ott et al. 2019). With regard to locally advanced, recurrent or metastatic courses of disease, a putative therapeutic response to checkpoint inhibitors should be verified in prospective treatment studies.

Many studies aimed to determine the prognostic impact of PD-L1 expression on the patient’s survival. Regarding different cancer entities the prognostic value of PD-L1 is controversial (Wang 2019; Wang et al. 2017; Troiano et al. 2019). In this study, the CPS which combines the expression of tumor cells and immune cells, seemed to provide prognostic information for SCCV, while TPS and ICS did not correlate with the patient’s outcome. High CPS was associated to a significant shorter OS (*p* = 0.021), although high CPS failed to be correlated to the PFS (*p* = 0.157). By now the prognostic impact of PD-L1 in SCCV was only analyzed in a few studies. According to Sznurkowski et al., PD-L1 expression in immune cells indicates a better prognosis (Sznurkowski et al. 2017), whereas PD-L1 expression in tumor cells was associated to worse outcome by Hecking et al. (2017). In addition, focusing p16-negative cases only high PD-L1 expression tended to correlate with a worse OS in our study population (*p* = 0.071). Correlation of PD-L1 expression with HPV-negative SCCV and poor outcome was also reported by Hecking et al. (2017).

Concurring with the expected frequency, p16-positivity was found in 39% of the cases, indicating a HPV-associated carcinogenesis. In this study, the status of p16 turned out to be an independent positive prognostic factor for SCCV. According to the 4th WHO classification, the prognostic impact of the HPV status is still considered to be unclear (Del Pino et al. 2013). However, indications increase that HPV-association is a positive predictive factor for SCCVs (Lee et al. 2016; Horne et al. 2018). Regarding oropharyngeal squamous cell cancer, several studies could verify p16 as a reliable surrogate marker for HPV-association (Prigge et al. 2017; Ma and Lewis 2012; Tan et al. 2016). However, it seems to be of interest that overexpression of p16 was also found in single cases of HPV-negative SCCV (Sznurkowski et al. 2016). Usually positive p16-staining correlates with oncogenic HPV infection by inactivation of the retinoblastoma protein via the viral E7 oncoprotein, but there are also HPV-independent mechanisms resulting in p16 expression (Riethdorf et al. 2004). Additionally, there may be differences in the definition of p16-positive. Only a strong “block” staining should be considered positive.

Finally, our data indicate that expression of PD-L1 is independent from the status of p16 in SCCV. Similar results have been published by Choschzick et al. (2018) and Thangarajah et al. (2019), indicating that PD-L1 expression is HPV-independent in SCCVs. These findings are supported by investigations on oropharyngeal SCCs that could show no correlation of the HPV-status and PD-L1 (Kim et al. 2016; Hong et al. 2019). Otherwise, PD-L1 was correlated to HPV-negativity in SCCVs too (Hecking et al. 2017). Thus, additional functional studies are needed to clarify the role HPV plays in PD-L1 induction.

TILs are discussed to be a reflecting indicator to the immune response of cancer. High numbers of TILs are believed to be associated with a better prognosis for many tumor entities (Badalamenti et al. 2018). Expression of PD-L1 was strongly associated to the amount of TILs in our cohort of SCCVs. This is reported for several different cancer types and is based on model that TILs may mediate PD-L1 expression in tumor cells by interferon release (Abiko et al. 2015; Badalamenti et al. 2018). However, TILs were not of prognostic importance in our cohort of vulvar SCCs. Further characterization of the infiltrate of TILs in SCCV will be needed to validate TILs as promising marker to select patients who may benefit from specific immunologic treatments.

In summary, our results show that PD-L1 positivity is a frequent finding in SCCV. PD-L1 immunoreactivity seems to be independent from p16 status and tends to indicate a worse outcome whereas p16 positivity turned out to be an independent positive prognostic marker.

## Electronic supplementary material

Below is the link to the electronic supplementary material.

Supplementary file1 (TIFF 21731 kb)

Supplementary file2 (DOCX 13 kb)

Supplementary file3 (TIFF 108 kb)
