# Supplementary figures and images for: PD-L1 expression and survival in p16-negative and -positive squamous cell carcinomas of the vulva
Source: J Cancer Res Clin Oncol. 2020 Feb 5;146(3):569–77. doi: 10.1007/s00432-020-03126-9 (PMC7040065; doi:10.1007/s00432-020-03126-9)

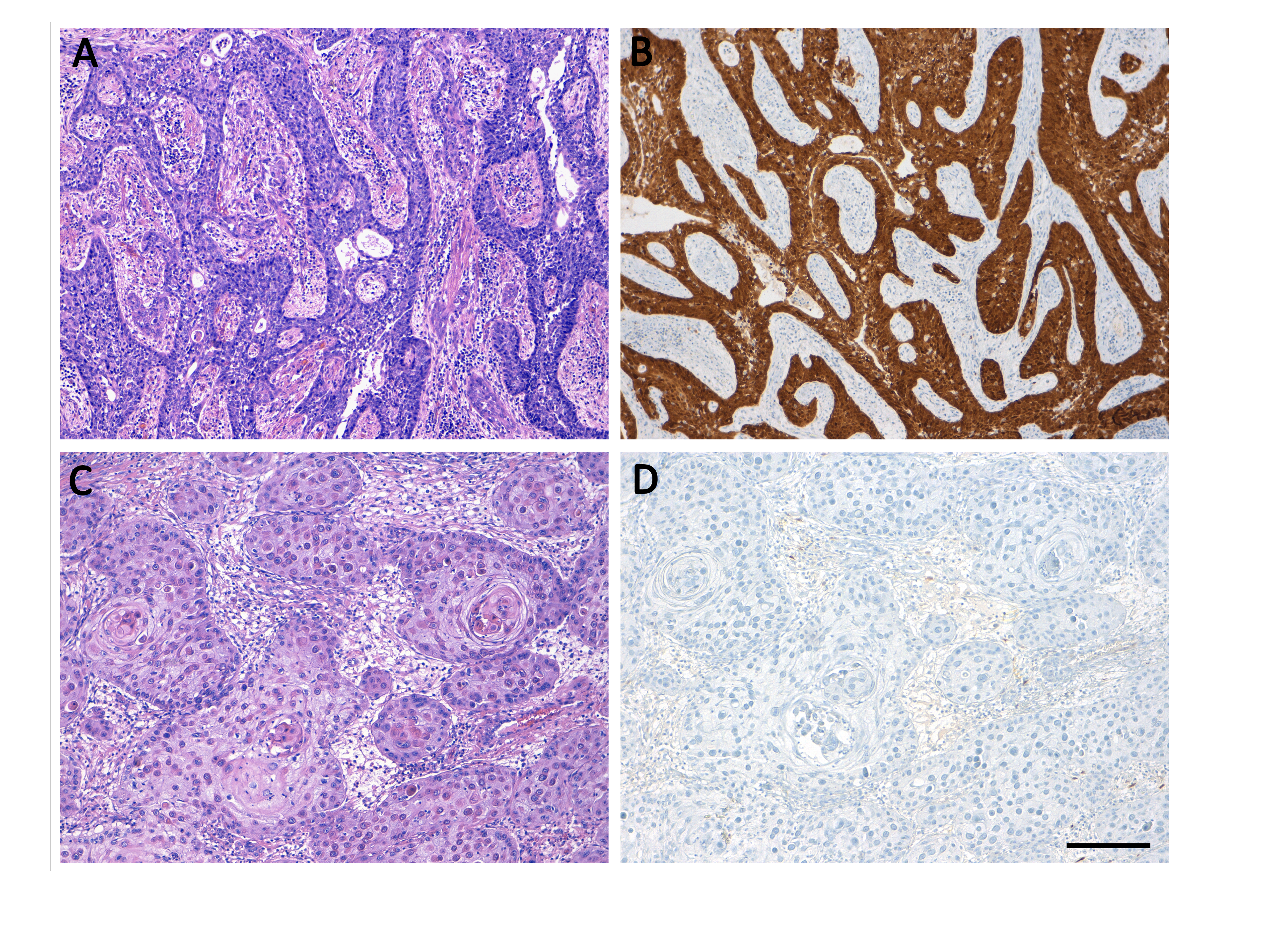

Supplement: Supplementary file 1 — Supplementary file1 (TIFF 21731 kb) [file 432_2020_3126_MOESM1_ESM.tif]

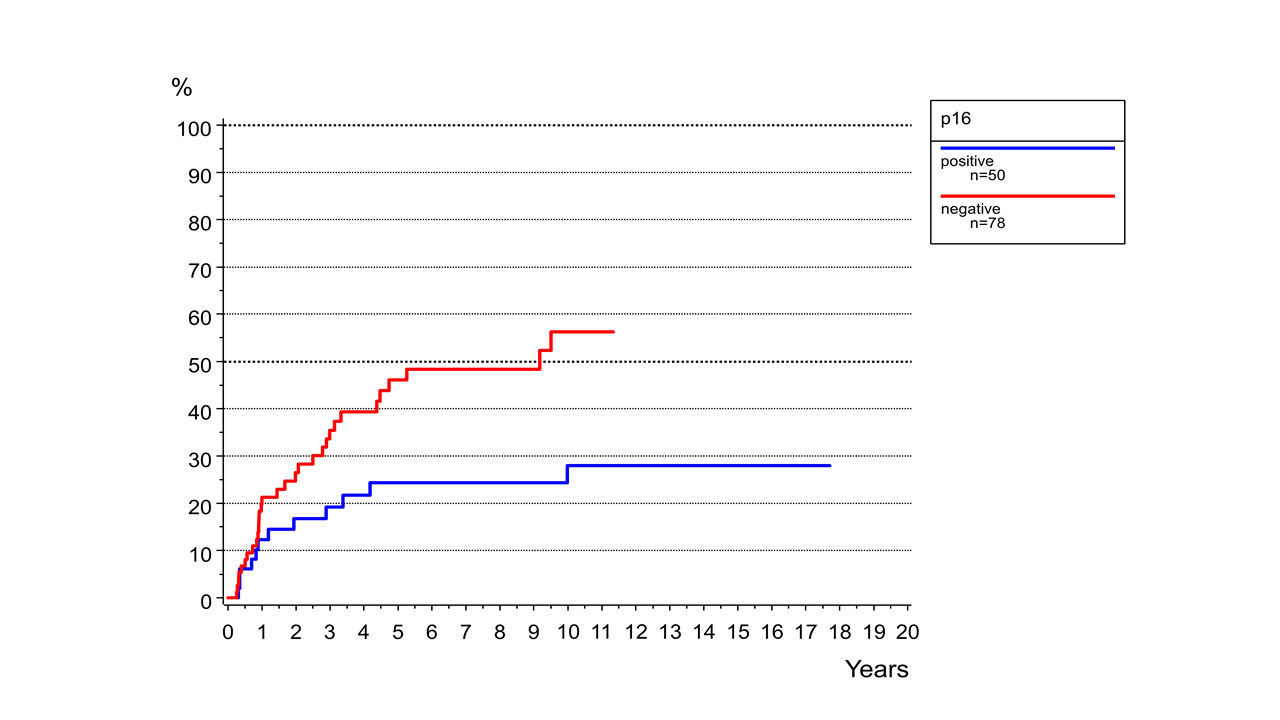

Supplement: Supplementary file 3 — Supplementary file3 (TIFF 108 kb) [file 432_2020_3126_MOESM3_ESM.tif]
