# Supplementary material for: PD-L1 expression and survival in p16-negative and -positive squamous cell carcinomas of the vulva
Source: J Cancer Res Clin Oncol. 2020 Feb 5;146(3):569–77. doi: 10.1007/s00432-020-03126-9 (PMC7040065; doi:10.1007/s00432-020-03126-9)
Supplement: Supplementary file 2 — Supplementary file2 (DOCX 13 kb) [file 432_2020_3126_MOESM2_ESM.docx]

Supplementary Tables

Supplementary Table 1a: Multivariate cox regression analysis with overall survival for TPS (n = 128)

| Variables | p-value | Hazard Ratio | 95% Confidence Interval |
| --- | --- | --- | --- |
| Age  (<70 versus ≥70) | <0.001 | 0.286 | 0.165 - 0.495 |
| Grading  (G1 versus G2/3) | 0.347 | 0.712 | 0.351 - 1.445 |
| FIGO  (I versus II-IV) | 0.005 | 0.403 | 0.212 - 0.763 |
| TPS  (<50% versus ≥50%) | 0.135 | 0.659 | 0.381 - 1.138 |

Supplementary Table 1b: Multivariate cox regression analysis with overall survival for ICS (n = 128)

| Variables | p-value | Hazard Ratio | 95% Confidence Interval |
| --- | --- | --- | --- |
| Age  (< 70 versus ≥ 70) | <0.001 | 0.311 | 0.182 - 0.530 |
| Grading  (G1 versus G2/3) | 0.210 | 0.641 | 0.319 - 1.285 |
| FIGO  (I versus II-IV) | 0.003 | 0.380 | 0.201 - 0.718 |
| ICS  (<10% versus ≥10%) | 0.414 | 0.831 | 0.553 - 1.295 |

Supplementary Table 2: Multivariate cox regression analysis with overall survival for CPS in p16-negative cases (n = 78)

| Variables | p-value | Hazard Ratio | 95%-Confidence Interval |
| --- | --- | --- | --- |
| Age  (<70 versus ≥70) | 0.009 | 0.430 | 0.230 - 0.807 |
| Grading  (G1 versus G2/3) | 0.213 | 0.593 | 0.261 - 1.348 |
| FIGO  (I versus II-IV) | 0.046 | 0.499 | 0.252 - 0.987 |
| CPS  (<50 versus ≥50) | 0.071 | 0.576 | 0.316 - 1.049 |

Supplementary Figure

Supplementary Figure: P16-positive squamous carcinoma of the vulva in A (HE) and B (p16) versus a p16-negative case in C (HE) and D (p16). Scale bar = 200µm for each picture
